# The expression of cerebrospinal fluid exosomal miR-630 plays an important role in the dysfunction of endothelial cells after subarachnoid hemorrhage

**DOI:** 10.1038/s41598-019-48049-9

**Published:** 2019-08-08

**Authors:** Leitao Sun, Wensheng Zhang, Zefu Li, Meng Li, Jiwei Guo, Hongyan Wang, Xiaohong Wang

**Affiliations:** 1 Department of Neurosurgery, Binzhou Medical University Hospital, Binzhou, Shandong China; 2 Cancer research institute, Binzhou Medical University Hospital, Binzhou, Shandong China; 3 Department of Thyroid and Breast Surgery, Binzhou Medical University Hospital, Binzhou, Shandong China

**Keywords:** Focal adhesion, Blood-brain barrier, Stroke

## Abstract

The purpose of this study was to evaluate the relationship of brain microvascular endothelial cell (BMECs) function and the exosomal miR-630 expression after subarachnoid hemorrhage (SAH). We evaluated the effects of blood cerebrospinal fluid (BCSF) on proliferation of BMECs by MTT at 0, 1, 3, 7 and 12 days and performed cell cycle analysis after BCSF treatment for 48 h. The expression of endothelial adhesion molecules (ICAM-1, VCAM-1 and ZO-1) were detected by qRT-PCR and immunofluorescent staining after BCSF treatment. NO produced by BMECs was also evaluated by Griess assay. The expression of exosomal miR-630 was analyzed by qRT-PCR in BCSF treated cell cultu normal cell culture medium andre medium. We further compared the exosomal miR-630 of clinical patients between aSAH and normal hydrocephalus. The adhesion molecules expression was further detected after co-incubation with exosomes transfected by miR-630 mimics. We found that BCSF significantly reduced the cell vitality in a time-dependent manner (*p* < 0.05) and the growth inhibition ratio reached 78.34 ± 9.22% on the 12th day. BCSF induced cell cycle arrest in G0/G1 phase in BMECs (*p* < 0.01). The expression of ICAM-1, VCAM-1, ZO-1 and the NO produced by BMECs were markedly reduced following incubation with BCSF. Then we demonstrated that the expression of exosomal miR-630 was markedly reduced in the BCSF treated BMECs and the same phenomenon occurred in aSAH patients compared with normal hydrocephalus. The expression of ICAM-1, VCAM-1 and ZO-1 were then increased in BMECs cocultured with exosomes transfected by miR-630 mimics. In conclusion, the low expression of exosomal miR-630 in CSF was closely related to endothelial function in BCSF endothelial cell injury model and clinical patients.

## Introduction

Aneurysmal subarachnoid hemorrhage (aSAH) is a devastating form of hemorrhagic stroke with 30-day mortality between 33–45%. So far, the mechanism of brain injury after SAH is not completely clear. Previous studies have suggested that vasospasm is an important cause of prognosis in patients. However, the application of nimodipine and ET-1 antagonists haven’t achieved satisfactory therapeutic effects^1,2^. In recent years, most researchers have focused on the role of brain micro-circulation disruption in early brain injury after SAH. The potential mechanisms include inflammation, oxidative stress injury, platelet activation, long-term vasoconstriction and endothelial cells (ECs) apoptosis^3^. The main structural and functional basis for brain microcirculation is the blood-brain barrier (BBB), which is composed of vascular endothelial cells, endothelial cell-cell junctions, basement membrane and astrocytes. Under physiological conditions, integrity of the structure and function of ECs maintain the integrity of BBB, but change with SAH. Damaged microvascular endothelial cells trigger a series of cerebrovascular injuries, which in turn exacerbate brain microvascular endothelial cell (BMEC) injury. However, the precise relationship between BMECs and brain injury remains unclear. Recent studies have shown that patients with BMEC dysfunction, tight junction degradation, disrupted cytokine secretion and electrolyte balance are vulnerable to brain micro-circulation^4–6^. Therefore, the early and accurate assessment about the degree of BMEC injury is critical for SAH.

Exosomes exist and circulate in cerebrospinal fluid (CSF), containing and transporting a broad spectrum of nucleotide and protein species. Importantly, the exosomal miRNA expression levels could reflect the physiology, pathology and function of cells^7^. Many studies have focus on the changes of exosomal miRNA expression in peripheral blood after vascular disease and acute ischemic stroke^8,9^. However, we consider that exosomal miRNAs in CSF are better protected from degradation, better reflect the functional indicators of endothelial cells. Although so far, little research is known about the role of exosome miRNAs in SAH pathogenesis.

Studies on aSAH patients showed that 13 miRNAs changes largely reflected differences between non-SAH and SAH groups, and a number of miRNAs, such as miR-27a-3p, miR-516a-5p, miR-566, and miR-1197 were exclusively different between vasospasm and non-vasospasm SAH groups^10^. Another clinical study showed that 66 miRNAs were significantly increased in SAH patients compared with neurologically healthy patients^11^. Recently, a study showed that exosomal miR-630 was a putative key mediator of vascular function and cardiovascular disease risk in children with underlying obstructive sleep apnea and/or obesity^12^. In previous studies, miR-630 had been reported to act as either an oncogene or a tumor suppressor in different cellular contexts by exerting pleiotropic functions and affecting cell proliferation, metastasis and apoptosis^13^. In our preliminary experiment, the expression of miR-630 in CSF exosomes of SAH patients was significantly decreased compared with chronic hydrocephalus patients. This result was inconsistent with the current studies on total miRNAs in CSF after SAH.

In this study, we further evaluated the effects of BCSF on BMECs and investigated the expression of exosomal miR-630 from cell culture medium and clinical patients following SAH.

## Materials and Methods

This investigation was organized in full accordance with the Helsinki Declaration^14^. Approval of the study protocol (ethical vote number 2019-013-01) was obtained from the Binzhou Medical University Hospital Ethics Committee and all participants have given their written informed consent for participation and the use of their respective data for research purposes.

### Cell culture

Human brain microvascular endothelial cells (BMECs, Cell Systems, Kirkland, WA, USA) were cultured in DMEM medium and incubated at 37 °C and 5% CO_2_.

### Preparation of the BCSF

Normal cerebrospinal fluid were purchased from United States BIOLOGical (Catalog: 3052). BCSF was prepared following Foley’s and u’sds^15,16^. Briefly, donor patient arterial blood and normal cerebrospinal fluid under aseptic condition were mixed with 1:1 ratio and incubated in a 37 °C water bath for 24 h. Then, the mixed samples were centrifuged at 10,000 × g for 20 min at room temperature. The supernatant was collected and stored at 4 °C. Before use, the above centrifuge matched with the DMEM medium by a 1:2 ratio.

### The effects of BCSF on cell proliferation

The BMECs were inculcated on board or cultivation bottles to nearly confluence of monolayer, then replaced the serum free medium for 24 hours. The cells were randomly divided into normal control group and BCSF different stimulus days groups (0, 1, 3, 7, 12 days). The normal control group was added normal cerebrospinal fluid mixed with DMEM medium at the ratio of 1:2. The BCSF stimulus group cells were cultured with the BCSF medium as described above for 0, 1, 3, 7, 12 days respectively. Both these cells were respectively seeded into a 96-well plate in triplicate at a density of 1 × 10^4^ cells/well. Empty wells were set in 96-well plate as blank control, while cell-free wells with reagent were used as negative control. After incubation, 5 mg/ml MTT (20 µL per well) was added. Cells were subsequently incubated at 37 °C for 4 h, after which 150 µL DMSO was added and the cells were incubated for another 15 min. Absorbance of each well was measured at 490 nm with a micro-plate reader (Bio-Rad Laboratories, Inc). Cell viability curve was subsequently plotted.

### The effects of BCSF on cell cycle

Cells were plated at a density of 5 × 10^4^ cells/well in 2 mL of complete DMEM mediumin 6-well plates, and allowed to grow for 24 h until 70% confluence. To achieve synchronization, cells were starved in serum-free medium for 24 h. Upon return to regular growth medium, cells were treated or untreated by BCSF. After 48 h of culture, cellular DNA content of 1 × 10^4^ cells from each sample was determined by cell cycle kit (KeyGen Biotech Co, Ltd). Cell cycle phase distribution was analyzed with the ModFit LT 3.2 software and samples were analyzed by BD FACS Calibur (BD Bioscience USA).

### The influence of BCSF on NO produced by BMECs

In order to evaluate the effect of BCSF on NO production, these stable metabolites of NO, nitrite and nitrate were measured by reduction of nitrate with reduced nicotinamide adenine dinucleotide phosphate (NADPH)-dependent nitrate reductase combined with detection with the acidic Griess reagent. The normal control group was added to normal cerebrospinal fluid, and the BCSF stimulus group joined the BCSF. 50 μL plasma sample/sodium nitrite standard was added to 96-well plate followed by 40 μL of conversion buffer with nitrate reductase and 10 μL of NADPH solution. Plates were shaken and incubated for 45 minutes at room temperature. Total nitrite in the sample was determined by Griess assay. Absorbance was measured at 540 nm. The total concentration of nitrite in sample was calculated from the nitrite standard curve. This gives the amount of nitrite stoichiometrically converted from nitrate, plus the originally present nitrite^17^.

### The effect of BCSF on BMECs were detected by qRT-PCR and immunofluorescent staining

To detect the effect of BCSF on BMECs, the expression of ICAM-1, VCAM-1 and ZO-1 were analyzed by qRT-PCR and immunofluorescence. qRT-PCR was performed after BCSF treatment for 24 h. The primer sequences were designed and supplied from Sangon BiotechCo., Ltd. (Shanghai, China) as follows: ZO-1 (forward: 5′-AGTGCCGCCTCCTGAGTTTG-3′, reverse: 5′-CCATCCTCATCTTCAT CATCTTCTACAG-3′), VCAM-1 (forward: 5′-GAGGATGGAAGATTCTGGAATT TACG-3′, reverse: 5′-ATCACTAGAGCAGGTCATGTTCAC-3′), ICAM-1 (forward: 5′-GCCACTAACAATCACGCATAATG-3′, reverse: 5′-TGCTCACTGTAGT CCCTTCTG-3′) and β-actin (forward: 5′-CCTGGCACCCAGCACAAT-3′, reverse: 5′-GGGCCGGACTCGTCATAC-3′). The expression and intracellular localization were also analyzed using immunofluorescence. Following 72 h incubation with BCSF, cells were washed with DMEM medium and fixed with 4% (w/v) paraformaldehyde in PBS for 20 min at room temperature and then washed again with PBS. The samples were blocked with 3% (w/v) bovine serum albumin in PBS for 30 min to block unspecific binding sites, and followed by overnight incubation at 4 °C with ICAM-1 (1:250), VCAM-1(1:100) and ZO-1(1:400). Alexa 488 was used as secondary antibodies (1:400, 2 mg/ml; Proteintech Group, CHI, USA). Images were captured by fluorescent microscopy.

## Exosome Isolation and Characterization

Exosomes were isolated from normal cell culture medium and BCSF treated cell culture medium using the Total Exosome Isolation Kit according to the manufacturer’s protocol (Life Technologies, Carlsbad, CA). To reduce the influence of exosomes in FBS, FBS was depleted of exosomes by ultracentrifugation at 200,000 g, 4 °C for 16 h. Supernatants were filtered through a 0.22 μm sterile filter and subsequently mixed with serum free media to prepare exosomes depleted cell culture media containing 10% FBS. Cells were grown in exosomes depleted culture media up to 80% confluence and we harvest exosomes. Briefly, culture medium was centrifuged at 2000 g for 20 minutes to remove cell/debris. The supernatants were collected and 0.2 volume of the Total Exosome Isolation Reagent was added. The mixtures were incubated at 4 °C for 30 minutes followed by centrifugation at 10,000 g for 10 min, and pellets were resuspend by PBS. Exosomes were analyzed by transmission electron microscopy (TEM) using negative staining. A drop of exosomes (about 10 μL) was added on copper grid for 1 min, dried at 65 °C and observed on a HT7700 transmission electron microscope (HITACHI, Japan) equipped and operated at an acceleration voltage of 80 kV. Images were taken using a Gatan CCD (Gatan, Inc, US). The size distribution was detected by a nano-ZS90 analyzer (Malvern, Worcestershire, UK) after diluted 10 times. The final pellets were used directly or re-suspended in PBS or SDS sample buffer and stored at −80 °C for follow-up test.

### Exosomal miR-630 qRT-PCR analysis in BCSF treated BMECs

Total RNAs that include small RNA fraction from exosomes isolated from normal cell culture medium and BCSF treated cell culture medium were isolated using SeraMir™ Exosome RNA Amplification Kit (System Biosciences) according to the manufacturer’s instructions. cDNA template for detecting miRNAs was generated using the Taqman MicroRNA Reverse Transcription Kit (Thermo Fisher Scientific) followed by qPCR using the TaqMan MircroRNA Assays for miR-630 with TaqMan Fast Advanced Master Mix for fluorescence detection. All samples were performed in triplicate. The results were analyzed with the Applied Biosystems SDS software (Applied biosystems, Waltham, MA) for Ct values and melting curve analyses. Relative expression levels were calculated via the 2^−ΔΔCt^ method. miR-16 and U6 were used for relative quantification and results were normalized to miR-16 expression^18,19^.

### Patient CSF collection and exosomal miRNA-630 qRT-PCR analysis

This study was carried out in accordance with approval from the Binzhou Medical University Hospital Ethics Committee. Informed consent was obtained for the collection of samples and subsequent analysis. All patients were enrolled at the Binzhou Medical University Hospital between February 2018 and November 2018. Men and women 40–55 years of age were eligible to participate in the study. The pilot study group comprised 8 patients (5 females and 3 males) and 4 control patients with normal pressure hydrocephalus (Delayed normal hydrocephalus secondary to SAH). The study was carried out in accordance with the Declaration of Helsinki.

CSF was collected via lumbar puncture from 8 aSAH patients at days 3 post hemorrhage. Inclusion criteria: (i) Onset of new neurological signs of aSAH within 48 h at the time of evaluation. (ii) Clinical signs consistent with the diagnosis of aSAH including severe thunderclap headache, cranial nerve abnormalities, decreased level of consciousness, meningismus and focal neurological deficits. (iii) Computed tomography demonstrates aSAH (Fisher 3 grade and Hunt-Hess 2 or 3 grade). (iv) Cerebral angiography reveals the presence of saccular aneurysms in a location that explains the aSAH. (v) Treatment of cerebral aneurysm must be carried within 48 h of symptom onset. (vi) Accepted treatments of aneurysms include surgical clipping or endovascular embolization. (vii) Lumbar puncture was performed to release BCSF within 72 h. Exclusion Criteria: (i) No demonstrable aneurysm by cerebral angiography. (ii) Evidence of traumatic, mycotic, or fusiform aneurysm by cerebral angiography. (iii) Hunt and Hess scale of 4 or greater and Fisher scale of 0–1. (iv) Severe prior physical disability that precludes evaluation of clinical outcome measures. (v) Contraindications for lumbar puncture are present.

4 control patients with normal pressure hydrocephalus (Delayed normal hydrocephalus secondary to aSAH). Inclusion criteria: (i) aSAH for more than 1 month. (ii) Lumbar puncture pressure is in the normal range. (iii) Lateral ventriculoperitoneal shunt surgery is required. Exclusion criteria: (i) Cardio-cerebrovascular disease occurred again within 1 month; (ii)Immunological vascular inflammatory lesions; (iii) Clinical manifestations of high cranial pressure.

Following collection, the CSF was immediately frozen at −80 °C and was not subjected to centrifugation. Analyzing only the CSF supernatant for miRNAs levels would confound trends over time, as cells (especially leukocytes and red blood cells) in the CSF are known to lyse and may release miRNAs. The method of patient CSF exosomal miR-630 qRT-PCR analysis was the same as the front.

### Specific miR-630 mimics ***in vitro***

Exosome transfections were performed using the Exo-Fect™ Exosome Transfection Reagent as described by the manufacturer’s protocol (cat# EXFT20A-1; System Biosciences, Inc. SBI, Mountain View, CA, USA). The transfected exosome pellets were suspended in 300 µL PBS, and used in the ECIS system for the miRNAs mimics of interest (75 µL were added to approximately 5 × 10^5^ cells/well in a 12-well culture plate grown in exosome-depleted FBS, and reaching equivalent exosome concentrations across conditions^20^. miR-630 mimics were purchased from Life Technologies (Grand Island, NY, USA). The specifically desired increase or decrease in miR-630 content was verified using qRT-PCR.

### Effect of miR-630 mimics in BMECs

To further explore the effects of co-incubation of BMECs with exosomes transfected by miR-630 mimics, the expression of endothelial adhesion molecules (ICAM-1, VCAM-1 and ZO-1) were analyzed by western blot, and NO production was also evaluated according to the previous protocol. BMECs grown to 50% confluency were exposed to transfected exosomes in exosome free FBS containing complete endothelial cell medium for 48 h followed by cell lysis using Mem-PER™ Eukaryotic Membrane Protein Extraction Reagent (Thermo Fisher Scientific). Using β-actin as an internal reference, the membrane was subsequently incubated at 4 °C overnight with primary antibodies against ICAM-1, VCAM-1 and ZO-1 diluted 1:2000 and β-actin diluted 1:5000 (Proteintech Group, CHI, USA), and enhanced chemiluminescence (ECL) plus kit (Millipore, America) was applied for visualization.

### Statistical analysis

Data are expressed as mean ± standard deviation (mean ± SD). Statistical analysis was performed with the Student t test or one-way ANOVA with Bonferroni correction for multiple comparisons following a normal distribution. All other data analysis was performed with standard statistical software GraphPad Prism, version 8 (GraphPad, LaJolla, CA). A *p* value < 0.05 was considered statistically significant.

## Results

### The effect of BCSF on the viability of BMECs

**Figure 1:**
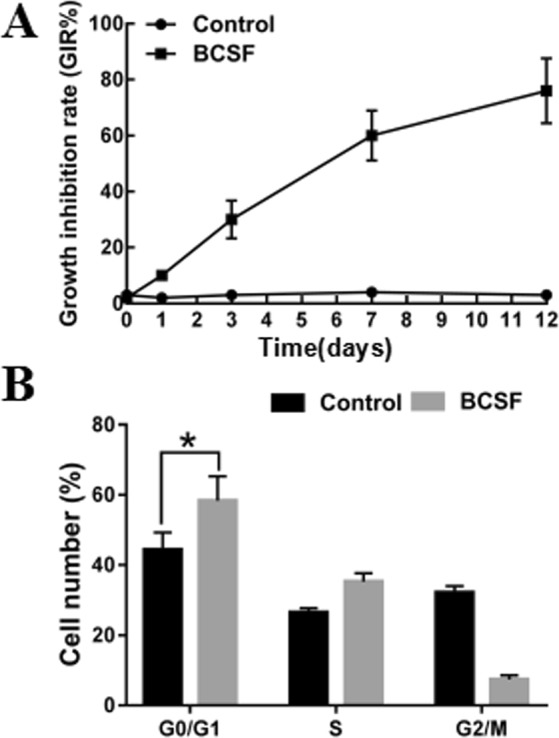
Effects of BCSF on the viability of BMECs. (**A**) MTT assay was performed after BMECs were treated with BCSF for 0d, 1d, 3d, 7d, 12d (*p* < 0.05). (**B**) Flow cytometry results showed that BCSF induced G0/G1 phase cell cycle arrest in BMECs (p < 0.01).

The MTT assay showed that the cell vitality of BMECs was significantly reduced after incubated by BCSF in a time-dependent manner (Fig. 1A) (*p* < 0.05). On day 12th, the growth inhibition ratio reached 78.34 ± 9.22%. Further, we performed cell cycle distribution analysis by flow cytometry. As shown in Fig. 1B, BCSF induced cell cycle arrest in G0/G1 phase and the percentage of G0/G1 phase was increased by (14.32 ± 4.28)% and G2/M phase was significantly decreased (*p* < 0.01) (Fig. 1B).

### The effect of BCSF on BMECs

**Figure 2:**
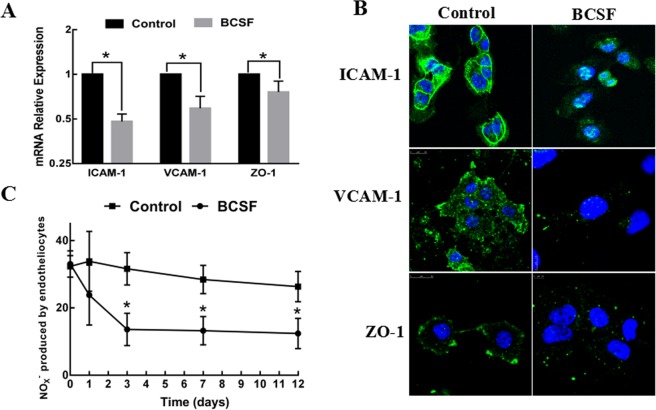
The effect of BCSF on endothelial function. (**A**) The mRNA expression of ICAM-1, VCAM-1 and ZO-1 was markedly reduced in BMECs treatment with BCSF for 24 h (*p* < 0.05). (**B**) The immunofluorescent staining showed that the BCSF group had weak signal of the tight junction protein ZO-1and the adhesion molecules ICAM-1, VCAM-1on cell membrane surface. (**C**) The NO_X-_ production was also significantly decreased after BCSF treatment, the greatest reduction especially in the 3rd day.

The effect of BCSF on the expression of tight junction protein ZO-1 and the adhesion molecules (ICAM-1, VCAM-1) were investigated by western blot. As shown in Fig. 2A, BCSF treatment for 24 h markedly reduced the mRNA expressions of ICAM-1, VCAM-1 and ZO-1 in BMECs. The immunofluorescent staining further showed BCSF group had weaker signal of the tight junction protein ZO-1 and the adhesion molecules ICAM-1, VCAM-1 on cell membrane surface (Fig. 2B). Concentration of NO by radioimmunoassy showed the NO produced by BMECs was also significantly decreased from 35.63 ± 4.78 μmol/L to 11.42 ± 2.66 μmol/L after BCSF treatment. As the extension of incubation time, NO production significantly declined, especially in the 3^rd^ day had the greatest reduction (Fig. 2C).

### Morphological and biochemical characterization of the exosomes

**Figure 3:**
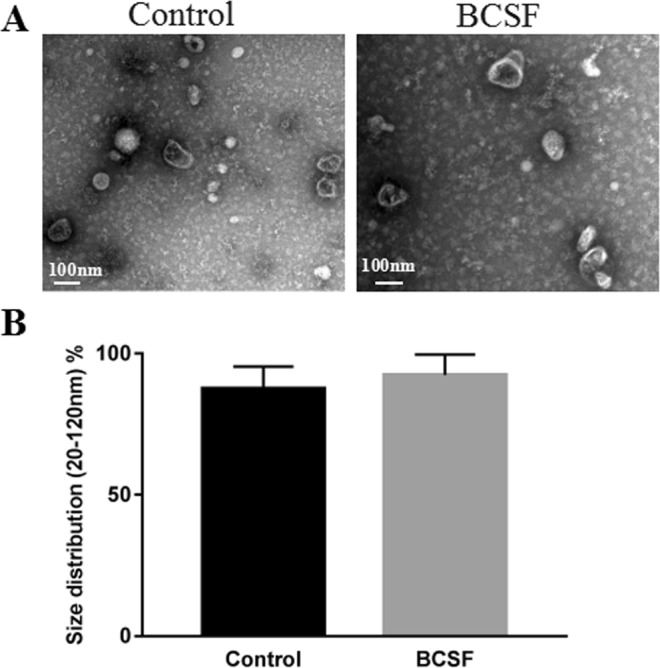
Morphological characterization of the exosomes. (**A**) Representative transmission electron microscopy image of normal cell culture medium and BCSF treated cell culture medium derived exosomes, showing a typical “saucer-like” morphology (scale bar, 100 nm). (**B**) Analysis of exosome size indicated similar particle size distribution between control group and BCSF group and >85% isolated exosomes displayed a diameter of 20–120 nm.

TEM analysis showed that the nanovesicles isolated from normal cell culture medium and BCSF treated cell culture medium were morphologically homogeneous with atypical round or cup shape appearance (Fig. 3A). Particle-size distribution of nano-AE PBS aqueous solution detected using nano-ZS90 (Malvern) showed that there were no significant deference between control group and BCSF group and >85% isolated exosomes displayed a size ranging from 20 to 120 nm in size (Fig. 3B).

### Exosomal miR-630 qRT-PCR analysis

**Figure 4:**
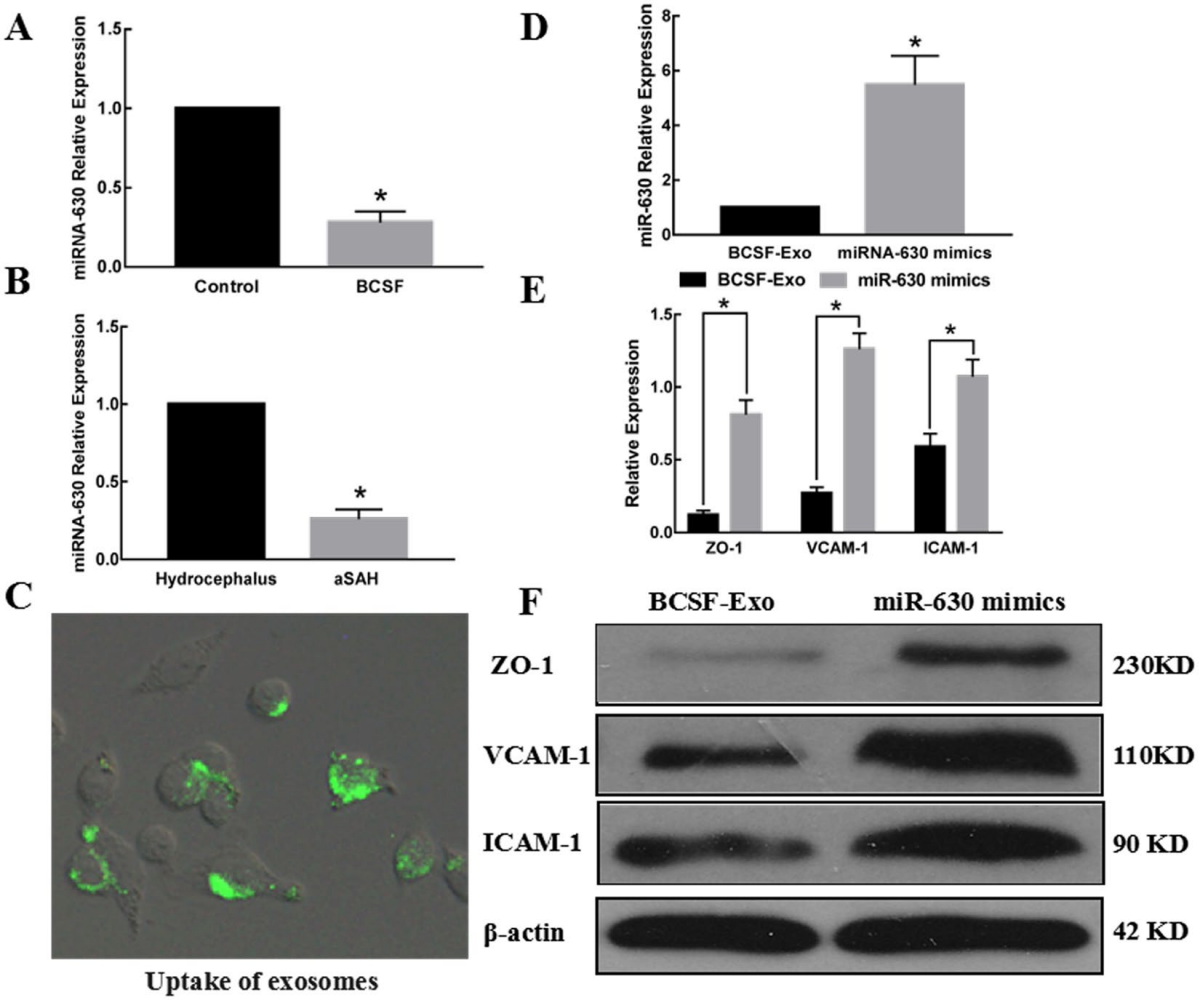
The influence of exosomal miR-630 on BMECs. (**A**) The expression of exosomal miR-630 was significantly lower in the BCSF treated group (*p* < 0.05). (**B**) The expression of exosomal miR-630 was also significantly reduced in SAH patients (*p* < 0.05). (**C**) The uptake of the fluorescently labelled miR-630 mimics transfected exosomes was evident in 90% BMECs after 12 h of incubation. (**D**) miR-630 was significantly increased after miRNA-630 mimics transfection (*p* < 0.05). (**E**,**F**) The expression of ICAM-1, VCAM-1 and ZO-1 in BMECs cocultured with exosomes transfected by miR-630 mimics was significantly increased compared to the control group (*p* < 0.01).

To determine whether exosomal miR-630 was differently expressed after BCSF treatment, total RNA was extracted from culture medium exosomes and qRT-PCR analysis was performed. As shown in Fig. 4A, the expression of exosomal miR-630 was significantly lower in the BCSF treated group (*p* < 0.05). We also determined the exosomal miR-630 expression in SAH and control patients. miR-630 showed 3.77-fold lower expression in SAH than in control patients (*p* < 0.05) (Fig. 4B).

### miR-630 over-expression in BMECs attenuate the decreased expression of adhesion molecules

Uptake of miR-630 mimics transfected exosomes by BMECs for up to 12 h was assessed using fluorescence microscopy after extensively washing the cells to remove any extracellular exosomes. A representative image of BMECs incubated with exosomes shown in Fig. 4C. In all cases we observed 90% of BMECs containing green fluorescent exosomes. The intracellular localization of these exosomes in the BMECs was mainly in the cell membrane and cytoplasm. miR-630 was significantly increased in BMECs co-cultured with exosomes transfected by miR-630 mimics(Fig. 4D). The expression of ICAM-1, VCAM-1 and tight junction protein ZO-1 in BMECs co-cultured with exosomes transfected with miR-630 mimics was significantly increased compared to control group by western blot and quantified by densitometry analysis (Fig. 4E) (*p* < 0.01). There were no significantly changes in production of NO.

## Discussion

This study is different from previous studies on the molecular mechanism of the effect of the components of hemorrhagic cerebrospinal fluid on the function of endothelial cells. We adopted the hemorrhagic cerebrospinal fluid and microvascular endothelial cell culture model of Foley and Zhi Liu *et al*., which was simple to make and easy to succeed. On the detection indexes, we detected the release of NO and the expression of ZO-1, ICAM-1 and VCAM-1. In the incubation process of BCSF, we found that the expression of ZO-1, ICAM-1 and VCAM-1 in BMECs was significantly reduced, which was similar to that of previous studies^12^. We successfully isolated the exosomes of cell culture medium and found that the exosomal miR-630 was down-regulated after BCSF treatment. Furthermore, the phenomenon showed a certain recovery after the application of specific miR-630 mimics. In addition, the miR-630 in patients with SAH was also down-regulated. Tight junctions regulate the paracellular flux of hydrophilic molecules across the BBB, and play a crucial role in the BBB, the ultra-structure of which appears as sites of apparent fusion involving the outer leaflets of the plasma membrane of adjacent ECs. In this study, we established an endothelial cell model *in vitro* following experimental SAH which was adopted in many studies. On the 3rd day of the experiment, the functional changes of BMECs were observed. Most studies believe that in the 3rd day after SAH, there’s severe vasospasm in the cerebral vessels, and the dysfunction of ECs is mainly manifested in the apoptosis of ECs. In addition, the decrease of ZO-1, ICAM-1 and VCAM-1 in BMECs reflects the endothelial cell barrier protection dysfunction. The expression of ZO-1, ICAM-1 and VCAM-1 reduced in BCSF group improved after intervention by miR-630 mimics transfection. These results indicated a significant role of exosomal miR-630 in activating tight junction between cerebral micro-vascular ECs which is the structural and functional base of BBB.

In recent years, many studies had focused on the microRNA in CSF after SAH and found that the miR-1224-3p and miR-1301 differently expressed in the hemorrhage group and the normal group, while miR-27a-3p, miR-516a-5p, miR-566, and miR-1197 showed significantly different in the cerebral vasospasm and non-vasospasm groups^21^. However, a clinical study found that the expression of miR-132-3p and 324-3p were changes after aneurysm rupture. Although the above studies only found a variety of miRNAs expression differences, the miRNA specific functions were not clear and the cell source couldn’t be determined. Khalyfa *et al*. have found the relationship of circulating plasma extracellular microvesicle miR-630 and endothelial dysfunction in obstructive sleep apnea children. The expression of miR-630 in serum exosomes could directly reflect the function of BMECs in pulmonary artery^12^. This is similar to the our findings. In our pre-experiment, the expression of miR-630 in CSF was not obviously different, however, the exosomal miR-630 was statistically significant. We assumed that the expression of miR-630 following SAH is multi-source and different in multiple central nervous system cells, and the expression of exosomal miR-630 in CSF could reflect the function of BMECs. Considering the complex processes during stroke, therapeutic targets should focus on preventive protection of the integrity of BMEC structure and function. Although more research is required to highlight the role of BMECs in stroke pathogenesis, additional strategies that target newly identified signaling pathways or molecules may offer a promising therapeutic approach to stroke.

The BCSF and microvascular endothelial cells co-culture model reflects the vasodilation and intercellular connectivity of endothelial cells in a certain extent, which is limited in the function of overall vascular endothelial cells. We will use the vascular culture model to further verify our conclusions in further studies. In addition, the secretion of exosomes is affected by many external factors, which still needs to be verified by large clinical samples. The regulation of miR-630 in exosomes and its downstream regulatory targets are still unclear which will be investigated in our further studies.

Our research has certain limitations. First of all, the co-culture model of BCSF and microvascular endothelial cells reflects the vasodilation and intercellular connectivity of endothelial cells in a certain extent, which is limited in the function of overall vascular endothelial cells. We will use the vascular culture model to further verify our conclusions in subsequent studies. Secondly, the secretion of exosomes is affected by many external factors, which still needs to be verified by large clinical samples. In addition, exosomes contain multiple miRNAs, and their interactions remain unclear. The expression regulation of miR-630 in the content and its downstream regulatory targets are still unclear which will be investigated in our further studies. In our further studies, we will explore the role of exosomes after aSAH through RNA-Seq and proteomic differences analysis and select healthy people as the control. Finally, we only studied the relationship between CSF exosome miRNA-630 and endothelial function. It is certain that CSF exosomes can be used to study the pathological changes of cerebral microcirculation. We look forward to more research in this area.

## Conclusions

BCSF reduced cell vitality and down-regulated the levels of exosomal miR-630 in BMECs. The expression of ICAM-1, VCAM-1, ZO-1 and the NO production were reduced after BCSF treatment which were reversed through over-expressing exosomal miR-630 in BMECs.
